# Long-term outcome and factors associated with restenosis after combination therapy of balloon angioplasty and stenting for symptomatic intracranial stenosis

**DOI:** 10.1186/s12883-022-03009-1

**Published:** 2022-12-13

**Authors:** Toshihiro Ueda, Satoshi Takaishi, Tomohide Yoshie, Noriko Usuki, Kentaro Tatsuno, Haruki Ohtsubo, Takashi Araga, Yasuyuki Kaga, Tatsuro Takada

**Affiliations:** 1 Department of Strokology and Neuroendovascular Treatment, Stroke Center, St. Marianna University Toyoko Hospital, Kosugi 3-435, Nakahara, Kawasaki, 211-0063 Japan; 2 Department of Practical Management of Medical Information, St. Marianna University School of Medicine, Kawasaki, Japan

**Keywords:** Intracranial artery stenosis, Balloon angioplasty, Stenting, Restenosis

## Abstract

**Background:**

The optimal treatment for intracranial artery stenosis (ICAS) has not been established. We retrospectively examined the initial and long-term outcomes associated with restenosis of a combination therapy of balloon angioplasty and stenting for symptomatic atherosclerotic ICAS.

**Methods:**

Consecutive patients who underwent balloon angioplasty and/or stenting for ≥ 70% ICAS between 2006 and 2020 were analyzed. Patients within 48 h of stroke onset were excluded. The following procedures were established as standards at our institution: (1) primary balloon angioplasty alone was initially performed; (2) stenting for insufficient dilatation, recoiling, or dissection was conducted; and (3) stenting was considered for restenosis. Intracranial ischemic and hemorrhagic complications within 30 days after treatment were used to evaluate periprocedural safety. Recurrent ischemic events, restenosis and restenosis related factors were used to be evaluate the long-term outcome.

**Results:**

A total of 160 patients were recruited. Initial treatment consisted of balloon angioplasty (*n* = 101) and stenting (*n* = 59). Intracranial complications within 30 days after treatment were ischemic in five (3.1%) and hemorrhagic in four patients (2.5%). The incidence of these complication was 3.1% in the stenting group and 2.5% in the balloon angioplasty group. The mean follow-up period was 53.9 months. Restenosis was found in 42 patients (26%). Recurrent ischemic events during follow-up were noted in 14 patients (8.8%), of which six patients had TIA and eight patients had ischemic stroke. Restenosis-associated factors included diabetes, coronary artery disease, percent stenosis after treatment, and balloon angioplasty in logistic univariate analysis. Multivariate Cox regression analysis showed that diabetes (HR: 2.084, CI: 1.039–4.180, *p* = 0.0386), length of lesion (HR; 1.358, CI: 1.174–1.571, *p* < 0.0001), and balloon angioplasty (HR: 4.194, CI: 1.083–16.239, *p* = 0.0379) were independent predictors for restenosis.

**Conclusion:**

Combination therapy of balloon angioplasty and stenting for symptomatic ICAS had a low perioperative stroke rate and may improve long-term outcome. Balloon angioplasty, diabetes, and length of lesion were significantly associated with restenosis.

## Background

Intracranial atherosclerotic stenosis (ICAS) is an important etiological factor for ischemic stroke, which accounts for 8 to 10% of patients with ischemic stroke in the United States, 18% in Japan, and 46% in China [1]. The recurrence rate in patients with symptomatic ICAS is high despite optimal medical treatment, although no treatment strategy has been established [2]. A recent cohort study with a follow-up of 2.8 years reported that the recurrence rate after medical treatment in patients with symptomatic ICAS was 14.9% [3].

The Stenting and Aggressive Medical Management for Preventing Recurrent Stroke in Intracranial Stenosis (SAMMPRIS) trial demonstrated the periprocedural stroke rate and death rate of intracranial stenting to be significantly higher (14.7%) than aggressive medical treatment (5.8%) [4]. However, as limitations of this trial, the rate of acute-phase patients was high and the operators had insufficient experience. The recent Wingspan Stent System Post Market Surveillance (WEAVE) trial, a prospective on-label study that used the Wingspan stent (Stryker, Kalamazoo, MI, USA), reported periprocedural stroke in 2.6% of patients [5].

Primary balloon angioplasty for symptomatic ICAS has been reported since the 1990s [6]. However, periprocedural complications and restenosis remain unresolved. Recently, primary balloon angioplasty without stenting was reported to have a lower periprocedural complication rate than stenting. Recent meta-analyses showed that the incidence of stroke and death within 30 days of submaximal angioplasty were 4.9% and 3%, respectively [7, 8]. The optimal endovascular treatment strategy for patients with symptomatic ICAS who do not improve after best medical treatment has not been established.

Although balloon angioplasty alone for symptomatic ICAS has been performed in Japan since the 1990s, the Wingspan stent has been used in selected patients after its approval in July 2014 [9]. The indication of the Wingspan stent has been limited to advanced dissection or acute occlusion after balloon angioplasty in Japan. We used selective balloon angioplasty and stenting based on arterial access and lesion morphology after the initial balloon angioplasty. The purpose of this study was to evaluate the initial and long-term outcomes of a combination therapy of balloon angioplasty and stenting for symptomatic ICAS at our institution. In addition, we investigated the factors increasing the risk of restenosis associated with endovascular treatment.

## Methods

### Patient selection

We retrospectively studied consecutive patients who underwent selective primary balloon angioplasty and stenting for symptomatic atherosclerotic intracranial artery stenosis from the prospectively acquired endovascular treatment databases of our institution between April 2006 and September 2020. Informed consent for the procedure was obtained from all patients. The study protocol was approved by the ethics review board of our institution.

Our inclusion criteria were: (1) patients with ≥ 70% stenosis of the main trunk of the middle cerebral artery (MCA), internal carotid artery (ICA), vertebral artery (VA), or basilar artery (BA) on cerebral angiography; (2) those with a history of a stenotic-lesion-induced transient ischemic attack (TIA) or non-disabling ischemic stroke (mRS ≤ 2); and (3) interval from the last TIA or ischemic stroke ≥ 72 h. Our exclusion criteria were: (1) residual stenosis immediately after recanalization therapy for acute major intracranial artery occlusion, (2) the presence of tandem extracranial lesions, and (3) pretreatment modified Rankin Scale score ≥ 3. We performed diagnostic imaging including cerebral angiography for patients with symptomatic ICAS, and initially start medical treatment. All patients received optimal medical treatment according to the guidelines at the time of treatment. For endovascular treatment, patients were selected in consideration of the indication criteria and exclusion criteria of our institution, and treatment is performed when consent is obtained from the patients and their families. Endovascular treatment was usually performed more than 2 weeks after the most recent onset.

Diagnostic criteria for atherosclerotic intracranial artery stenosis were as follows: (1) the absence of cerebral artery dissection on cerebral angiography, and (2) patients with ≥ 2 of the following vascular risk factors: hypertension, dyslipidemia, diabetes mellitus (DM), peripheral arterial disease, coronary artery disease, smoking, pre-existing atherosclerotic stenosis in other locations, and the presence of aortic plaque. Patients with ischemic stroke perform carotid echography, electrocardiogram, echocardiography, and transesophageal echocardiography to search for embolic sources in order to evaluate the etiology of ischemic stroke. Various blood tests and cerebrospinal fluid tests are performed to distinguish inflammatory diseases. If arterial dissection is suspected, in addition to cerebral angiography, MRA and CTA are repeated to check for morphological changes. These diagnostic studies were repeated when the stroke recurred.

The following procedures were established as standards for minimally invasive combination therapy at our institution: (1) as initial treatment, primary balloon angioplasty alone was performed, (2) stenting was conducted when balloon angioplasty lead to insufficient dilation or acute dissection, (3) stenting should be prioritized for restenotic lesions, and (4) coronary stents were used until July 2014, when the Wingspan stent was approved. Since then, Wingspan stents have been used. Two experienced neuro-interventionalists were responsible for all treatments.

In all patients, MRI/MRA was performed before treatment to evaluate infarcted cerebral foci. The ICA was classified into supraclinoid (C1-C2), cavernous (C3-C4), and petrous (C5) segments. In this study, perioperative complications within 30 days were evaluated as initial results and restenosis, and recurrent ischemic events during follow-up were evaluated as long-term outcomes. Furthermore, factors associated with restenosis and recurrent ischemic events were analyzed.

### Interventional techniques

All treatments were performed under venous anesthesia with propofol and local anesthesia at the site of femoral artery puncture. After a 6-Fr guiding catheter was inserted into the distal cervical internal carotid artery or distal vertebral artery, 5,000 units of heparin was intravenously administered. Percent stenosis was evaluated using the WASID method [10]. In addition, the lesion length and shape (eccentric or concentric) were assessed.

A 0.014-inch microguidewire was guided into a distal artery vessel across the target lesion, then a Gateway balloon catheter (Stryker, Kalamazoo, MI, USA) or Unryu balloon catheter (Kaneka Medics, Tokyo, Japan) was guided. The catheter measured 1.5 to 4.0 mm in diameter, slightly smaller than that of a normal artery distal to the site of stenosis, and 9 to 20 mm in length in accordance with the length of the stenotic site. The balloon catheter was dilated to a maximum atmospheric pressure of 6 atm by increasing the pressure by 1 atm every 10 s, and maintained for approximately 30 s. When the percent stenosis after dilatation was ≤ 50%, the procedure was regarded as successful. To evaluate the presence of elastic recoiling or dissection after dilatation, repeat angiography was performed several times. When acute dissection with distal blood flow deterioration or residual stenosis of 50% or more were observed, stenting was conducted. After treatment, neurological assessment was conducted and the patients were managed in the intensive care unit until the day after treatment. Anticoagulant therapy with heparin was discontinued.

### Periprocedural assessment

In all patients, MRI/MRA was performed the day after treatment. As procedure-associated complications within 24 h after treatment, we evaluated arterial dissection, vascular perforation, distal artery occlusion, acute occlusion, and acute in-stent thrombosis. Furthermore, we evaluated all ischemic events, systemic complications, and deaths within 30 days.

### Medical management

Dual antiplatelet therapy (DAPT) was performed from at least 1 week before treatment. Aspirin and clopidogrel were administered to most patients; however, cilostazol was administered to patients treated by Wingspan stent who had clopidogrel resistance, which was based on platelet aggregation evaluated by the VerifyNow P2Y12 Assay (Accumetrics Inc., San Diego, CA, USA). Single antiplatelet therapy (SAPT) was started the day after treatment in patients who underwent balloon angioplasty alone. When stenting was conducted, DAPT was continued for 6 months and switched to SAPT in the ischemic event- or restenosis-free patients. Risk factor control in the patients consisted of achieving systolic blood pressure less than 140 mmHg, the administration of statins for dyslipidemia, and blood glucose control for DM.

### Follow-up management

MRI and cerebral angiography were performed after 6 months to evaluate the presence of restenosis. Subsequently, MRI and MRA were conducted every 6 to 12 months. In this study, restenosis was defined as ≥ 50% stenosis. Recurrent ischemic events (cerebral infarction or TIA) were assessed in all regions in addition to the treated stenotic artery. Restenosis patients with recurrence of ipsilateral ischemic events were retreated with balloon angioplasty or stenting. Medical treatment was continued for asymptomatic restenosis patients. However, if the degree of restenosis progressed in a relatively short period of time, retreatment was considered. Additional treatment was indicated for the following patients: (1) those with ≥ 70% restenosis within 1 year after treatment, and (2) those with ≥ 50% restenosis with recurrent ischemic events. As long-term outcomes, restenosis and recurrent ischemic events were analyzed.

### Statistical analysis

Continuous variables were expressed as mean ± standard deviation. Continuous variables with non-normal distribution were summarized as medians (interquartile range). Categorical variables were presented as numbers and percentages.

Restenosis-associated factors included age, sex, vascular risk factors (hypertension, dyslipidemia, DM, coronary artery disease, and chronic kidney disease), stenotic artery (MCA, ICA, VA and BA), duration from last attack to treatment, percent stenosis before and after treatment, treatment procedure, length and shape of the stenotic lesion, maximum diameter of the balloon catheter or stent, and recurrence of ischemic events (TIA and ischemic stroke). Logistic univariate analysis was used for the factors that influenced the development of restenosis.　Hazard ratio (HR) and 95% confidence intervals (CI) were calculated using multivariate Cox proportional hazards regression models.　A *p*-value < 0.05 was considered statistically significant. All statistical analyses were performed with SPSS (version 9.4, IBM, USA).

## Results

The baseline characteristics of the patients (*n* = 160) are shown in Table 1. Their ages ranged from 39 to 88 years, with a mean of 67.6 years. There were 124 males and 36 females. The stenotic arteries consisted of the MCA in 68 patients (43%), ICA in 49 (31%), VA in 31 (19%), and BA in 12 (8%). Concerning ischemic symptoms, cerebral infarction was observed in 118 patients (74%) and TIAs were observed in 42 (26%). Seven out of 160 patients had multiple intracranial severe stenosis. In this study, only obvious symptomatic stenosis was treated. The median interval from an attack until treatment was 21 days. Aspirin at 100 mg and clopidogrel at 75 mg were administered to 133 patients (83%), and aspirin at 100 mg and cilostazol at 200 mg were administered to 27 (17%).

**Table 1:** Baseline characteristics of patients receiving endovascular treatment

Variables	Number (%)
Age, mean ± SD, y	67.6 ± 11.2
Sex, male, n (%)	124 (77.5)
Vascular risk factors	
Hypertension, n (%)	139 (86.9)
Dyslipidemia, n (%)	107 (66.9)
Diabetes mellitus, n (%)	61 (38.1)
Coronary artery disease, n (%)	29 (18.1)
Chronic kidney disease, n (%)	12 (7.5)
Medication	
Aspirin, n (%)	131 (81.9)
Clopidogrel, n (%)	140 (87.5)
Cilostazol, n (%)	27 (16.9)
Stenotic artery	
Middle cerebral artery, n (%)	68 (42.5)
Internal cerebral artery, n (%)	49 (30.6)
C1-2	10 (20.4)
C3-4	12 (24.5)
C5	27 (55.1)
Vertebral artery, n (%)	31 (19.4)
Basilar artery, n (%)	12 (7.5)
Symptom	
TIA	42 (26.2)
Ischemic stroke	118 (73.8)
Duration from last attack to procedure, median (IQR), day	21 (14—30)

*IQR* interquartile range

### Periprocedural results

As initial treatment, balloon angioplasty was performed in 101 patients (63%) and stenting was performed in 59 (37%) (Table 2). The treatment procedures were successful in all patients. The mean percent stenosis decreased from 78.2 ± 8.7% to 26.6 ± 13.3%. The length of lesion was 8.4 ± 3.3 mm. The morphology of the stenotic lesion was eccentric in 44.4% and concentric in 55.6%. The maximum diameter of the balloon catheter or stent used was 2.7 ± 0.9 mm. Wingspan and coronary stents were used in 43 and 16 patients.

**Table 2:** Procedural results and follow-up ischemic events

	Balloon angioplasty	Stenting		
		Coronary	Wingspan	Total
N	101	16	43	59
Age, Mean ± SD, y	67.0 ± 11.5	66.3 ± 8.3	69.5 ± 11.5	68.6 ± 10.8
Sex, male	76	12	36	48
Stenotic artery				
Middle cerebral artery	58	1	9	10
Internal carotid artery	25	9	15	24
Vertebral artery	9	6	16	25
Basilar artery	9	0	3	3
Length of lesion, Mean ± SD, mm	7.3 ± 2.3	9.4 ± 3.0	11.7 ± 4.5	10.7 ± 4.1
Morphology of lesion				
Eccentric	50	4	17	21
Concentric	51	12	26	38
Stenosis rate, Mean ± SD, %				
Pretreatment	78.8 ± 7.8	75.7 ± 9.1	74.2 ± 7.9	74.9 ± 8.4
Posttreatment	32.3 ± 12.9	14.7 ± 9.7	14.3 ± 6.5	14.5 ± 8.2
30-day perioperative complications				
Ischemic stroke	4 (4.0%)			1 (1.7%)
Perforator stroke	3	0	0	0
Embolic stroke	1	0	0	0
Stent thrombosis	-	0	1	1
Hemorrhagic stroke	2 (2.0%)			2 (3.4%)
SAH, dissection	1	0	0	0
SAH, perforation	0	0	1	1
Intracerebral hemorrhage	1	0	0	0
Intracerebral hemorrhage (HPS)	0	1	0	1
Restenosis	35 (34.7%)	3 (18.8%)	4 (9.3%)	7 (11.9%)
Recurrence of ischemic events	10 (9.9%)	2 (12.5%)	2 (4.7%)	4 (6.8%)
TIA	3	1	2	3
Ischemic stroke	7	1	0	1

*SAH* Subarachnoid hemorrhage, *HPS* Hyperperfusion syndrome

Perioperative intracranial complications within 30 days after treatment occurred in a total of nine patients (5.6%). Ischemic complications were noted in five patients (3.1%), consisting of three (3.0%) in the balloon angioplasty group and two (3.4%) in the stenting group. Perforator infarction was observed in three patients in the balloon angioplasty group and none in the stenting group. Furthermore, distal embolism (*n* = 1) and in-stent thrombosis (*n* = 1) were observed.

Hemorrhagic complications were observed in four patients (2.5%), consisting of acute arterial dissection-related subarachnoid hemorrhage (*n* = 1) and hyperperfusion syndrome-related intracerebral hemorrhage (*n* = 1) in the balloon angioplasty group, and subarachnoid hemorrhage related to distal vessel perforation (*n* = 1) and hemorrhagic infarction-related intracerebral hemorrhage (*n* = 1) in the stenting group. One patient with intracerebral hemorrhage after stenting for basilar artery stenosis died. The total number of patients with ischemic or hemorrhagic intracranial complications was five (5.0%) in the balloon angioplasty group and four (6.8%) in the stenting group,which were nearly identical. Comparison of stenosis improvement and perioperative complications between the coronary stent and the Wingspan stent groups showed similar results. On the other hand, restenosis and recurrent ischemic events tended to be slightly less common in the Wingspan stent group than in the coronary stent group (Table 2).

Recurrent ischemic events during follow-up were noted in 14 patients (8.8%), of which 6 patients had TIA and 8 patients had ischemic stroke. Eight of the 14 patients had ipsilateral ischemic events, of which 5 had TIA and 3 had ischemic stroke. Recurrence of these ischemic events occurred on average 43.1 ± 48.7 months after treatment without fatal strokes.

### Restenosis features

Posttreatment follow-up periods ranged from 6 to 182 months, with a mean of 53.9 months and median of 35 months. Of the 160 patients, restenosis was observed in 42 (26.3%) during follow-up periods. The incidence of restenosis was 5.8% per year. Overall restenosis occurred with a mean of 16.1 months and a median of 6.5 months, a mean of 15.7 months and a median of 6 months in the balloon angioplasty group, and a mean of 18.4 months and a median of 9 months in the stenting group. Restenosis was noted in 35 (34.7%) patients of the balloon angioplasty group and in seven (11.9%) of the stenting group.

An analysis of the factors that influence the development of restenosis is shown in Table 3. Significant factors associated with restenosis were DM (*p* = 0.0108), coronary artery disease (*p* = 0.0443), clopidogrel (*p* = 0.0471), posttreatment stenotic rate (*p* = 0.0039), and balloon angioplasty (*p* = 0.0025) in logistic univariate analysis. Recurrent ischemic events during follow-up were noted in six patients (14%) in the patients with restenosis and in eight (6.8%) in the patients without restenosis. There was no significant difference between the two groups in the recurrence of ischemic events.

**Table 3:** Examination of factors affecting restenosis by logistic univariate analysis

	Restenosis	Non-restenosis	*P* value
N(%)	42 (26.3)	118 (73.8)	
N	42	118	
Age			
Mean ± SD, y	65.9 ± 11.3	68.2 ± 11.2	0.2499
Median	69 (58–74)	70.0 (62–76)	
Sex			
Male	34 (27.4)	90 (72.6)	0.5335
Female	8 (22.2)	28 (77.8)	
Vascular risk factors			
Hypertension	37 (26.6)	102 (73.4)	0.7852
Dyslipidemia	27 (25.2)	80 (74.8)	0.6781
Diabetes mellitus	23 (37.7)	38 (62.3)	0.0108
Coronary artery disease	12 (41.4)	17 (58.6)	0.0443
Chronic kidney disease	3 (25.0)	9 (75.0)	0.9185
Medication			
Aspirin	36 (27.5)	95 (72.5)	0.4539
Clopidogrel	33 (23.6)	107 (76.4)	0.0471
Cilostazol	6 (22.2)	21 (77.8)	0.6026
Stenotic artery			0.7582
Middle cerebral artery	17 (25.0)	51 (75.0)	
Internal cerebral artery	16 (32.7)	33 (67.3)	
C1-2	4 (40.0)	6 (60.0)	
C3-4	2 (16.7)	10 (83.3)	
C5	10 (37.0)	17 (63.0)	
Vertebral artery	7 (22.6)	24 (77.4)	
Basilar artery	2 (16.7)	10 (83.3)	
Symptom			
Cerebral infarction	33 (28.0)	85 (72.0)	0.4098
TIA	9 (21.4)	33 (78.6)	
Last attack to treatment, day			
Mean ± SD	34.5 ± 33.3	30.7 ± 33.9	0.5412
Median	30 (11–50)	21 (14–30)	
Stenosis rate: pretreatment, %			
Mean ± SD	78.6 ± 9.2	78.1 ± 8.6	0.7357
Median	80 (70–85)	80 (70–80)	
Stenosis rate: posttreatment, %			
Mean ± SD	31.8 ± 11.1	24.7 ± 13.6	0.0039
Median	30 (25–40)	20 (15–30)	
Length of lesion, mm			
Mean ± SD	9.0 ± 3.6	8.1 ± 3.1	0.1404
Median	8.0 (7–11)	7.5 (6–9)	
Morphology of lesion			
Eccentric	23 (32.4)	48 (67.6)	0.1166
Concentric	19 (21.3)	70 (78.7)	
Max diameter of balloon/stent, mm			
Mean ± SD	2.5 ± 0.8	2.8 ± 0.9	0.0568
Median	2.3 (2.0–3.0)	2.5 (2.0–3.5)	
Primary procedures			
Balloon angioplasty	35 (34.7)	66 (65.3)	0.0025
Stenting	7 (11.9)	52 (88.1)	
Recurrence of ischemic events	6 (42.9)	8 (57.1)	0.1479
TIA	3 (50.0)	3 (50.0)	0.1965
Ischemic stroke	3 (37.5)	5 (62.5)	0.4626

Multivariate Cox regression analysis showed that DM (HR: 2.084, CI: 1.039—4.180, *p* = 0.0386), length of lesion (HR; 1.358, CI: 1.174—1.571, *p* < 0.0001), and balloon angioplasty (HR: 4.194, CI: 1.083—16.239, *p* = 0.0379) were independent predictors for restenosis (Table 4). Antiplatelet drugs were excluded from this multivariate analysis due to insufficient data on long-term oral medications.

**Table 4:** Multivariate Cox regression analysis for prediction factors of restenosis after endovascular treatment

	Hazard ratio	95% CI	*P* value
Age, y	1.008	0.970—1.047	0.7005
Sex			
Male	1.595	0.598—4.254	0.3509
Female	Reference	-	
Vascular risk factors			
Hypertension	1.818	0.508—6.507	0.3580
Dyslipidemia	0.899	0.439—1.841	0.7706
Diabetes mellitus	2.084	1.039—4.180	0.0386
Coronary artery disease	1.682	0.760—3.720	0.1996
Chronic kidney disease	0.719	0.200—2.583	0.6126
Vascular risk factors			0.2424
Middle cerebral artery	1.267	0.235—6.838	
Internal cerebral artery	1.511	0.276—8.268	
Vertebral artery	0.444	0.061—3.234	
Basilar artery	Reference	-	
Duration from last attack to procedure	0.993	0.982—1.004	0.2158
Stenosis rate: pretreatment	0.996	0.953—1.041	0.8656
Stenosis rate: posttreatment	1.009	0.979—1.041	0.5605
Length of lesion	1.358	1.174—1.571	< 0.0001
Morphology of lesion			
Eccentric	1.571	0.782—3.155	0.2043
Concentric	Reference	-	
Max diameter of balloon/stent	0.661	0.339—1.287	0.2232
Primary procedures			
Balloon angioplasty	4.194	1.083—16.239	0.0379
Stenting	Reference	-	
Recurrence of ischemic events			0.4396
TIA	2.733	0.579—12.903	
Ischemic stroke	1.130	0.292—4.375	

*CI* Confidence interval

### Long-term outcomes

Forty-five sessions of additional treatment for restenosis were performed in 37 patients. Of the 45 sessions, stenting and balloon angioplasty were performed in 24 (53%) and 21 (47%), respectively. The mean interval from initial treatment until additional treatment was 17.1 ± 24.4 months, with a median of 8 months.

**Fig. 1:**
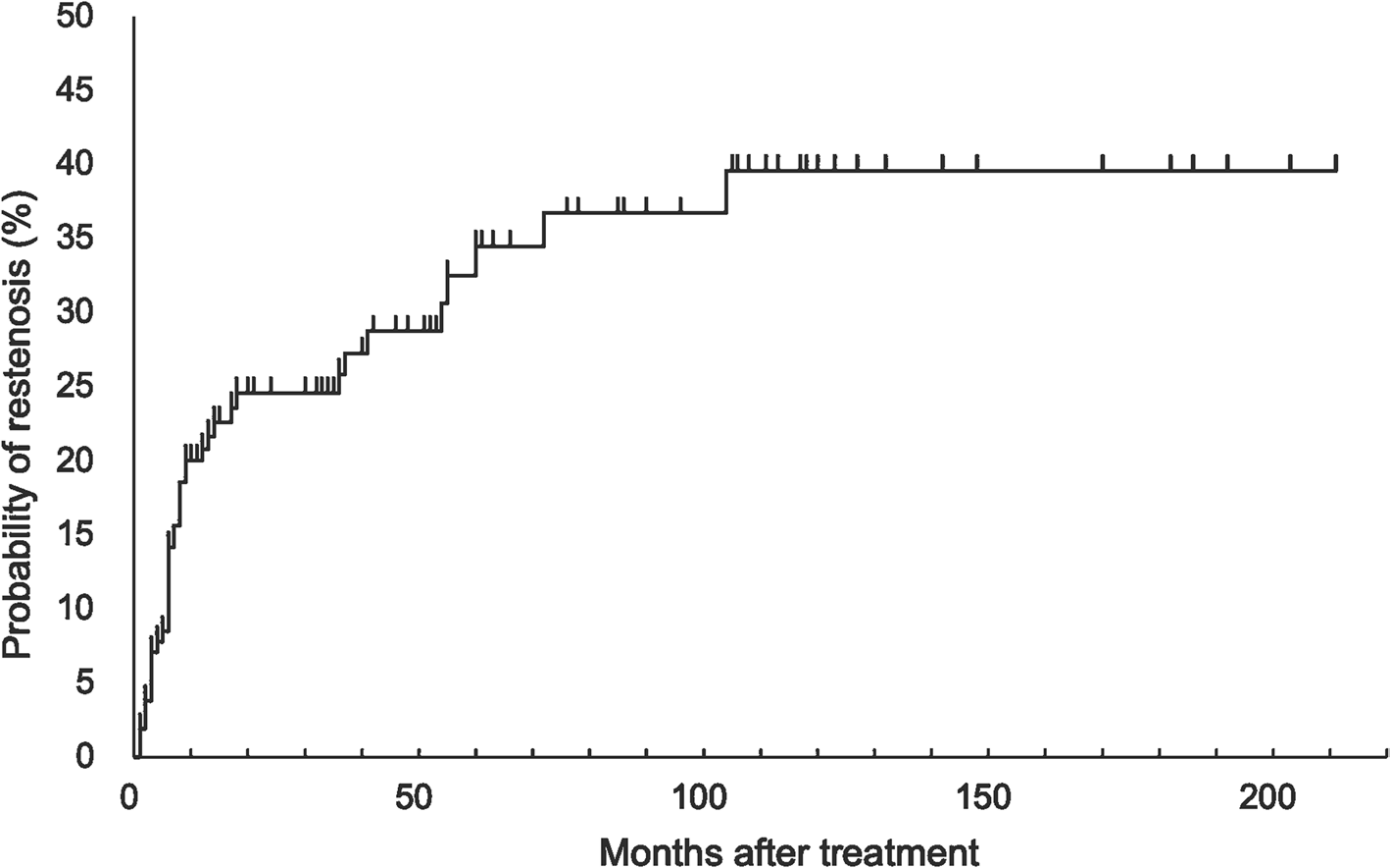
Kaplan–Meier curve for the cumulative probability of restenosis after endovascular treatment. Kaplan–Meier estimate of the cumulative restenosis rates in patients who received endovascular treatment for symptomatic intracranial stenosis. The 1-, 2-, 5-, and 10-year Kaplan–Meier rates (with 95% confidence interval) for restenosis after endovascular treatment among all 160 patients were 20.8% (15.0–28.4), 24.5% (18.1–32.7), 34.4% (25.6–45.3), and 39.6% (29.3–51.9), respectively

The Kaplan–Meier estimation of the cumulative probability of restenosis after endovascular treatment is shown in Fig. 1. The 1-, 2-, 3-, 5-, and 10-year Kaplan–Meier rates (with 95% confidence interval) for restenosis after endovascular treatment among all 160 patients were 20.8% (15.0—28.4), 24.5% (18.1—32.7), 25.8% (19.1—34.3), 34.4% (25.6—45.3), and 39.6% (29.3—51.9), respectively. The incidence of restenosis was highest within the first year after endovascular treatment.

## Discussion

Currently, there is no consensus regarding endovascular treatment strategies for intracranial stenosis. The problems of periprocedural complications and restenosis after treatment have not yet been resolved. We performed this retrospective study focusing on minimally invasive combination therapy tailored to individual patients rather than prioritizing intracranial stenting, and evaluated the periprocedural safety, long-term outcome, and factors associated with restenosis.

An increasing number of studies have recommended balloon angioplasty alone with minimally invasive techniques, such as submaximal dilatation [8]. The advantage of balloon angioplasty alone without stenting has been reported to be the limited damage to the vessel and relatively easy navigation. A meta-analysis showed that the incidence of stroke and death within 30 days was 4.9%, and that after Day 30 was 3.7% [11]. Another meta-analysis revealed that the incidences of stroke and death within 30 days and after 1 year were 3 and 5%, respectively [12]. The incidence of perioperative complications related to balloon angioplasty alone for symptomatic stenosis of the MCA was reported to be 4.2% [13]. In this study, the incidence of perioperative intracranial complications was 5.0% in the balloon angioplasty group and 6.8% in the stenting group.

The usefulness of stenting for symptomatic ICAS was not confirmed in a randomized controlled trial with medical treatment. Among ischemic complications, the incidence of perforator infarction was the highest, and basilar artery stenosis, advanced age, and DM were significantly associated with ischemic complications [14]. However, the results of treatment in a high-volume center through strict patient selection, excluding patients with acute ischemic stroke, were favorable. Wang et al. reported that the incidence of complications related to Wingspan stent insertion for MCA stenosis was 1.4% in the phase of skilled techniques [15]. Ma et al. reported that the incidence of perioperative complications in the Wingspan-stent-treated group was 4% [16]. In the WEAVE trial, the incidence of complications related to on-label procedures was 2.6% [5]. The conditions for on-label procedures in the United States included an age of 22 to 80 years, history of cerebral infarction (≥ 2 episodes), interval of ≥ 8 days from the onset of cerebral infarction, 70–99% stenosis, lesion length of ≤ 14 mm, and vascular diameter of ≥ 2 mm. On the other hand, the specifications of the Wingspan stent system, which was approved by the FDA in the United States in 2005, have not been modified to date, which raises an issue as a thick, hard delivery system must be guided.

There exists a lack of consensus regarding the best endovascular treatment strategy for symptomatic ICAS. To select an optimal treatment method, the site of the stenotic artery, lesion shape, and access route to the lesion are examined, and comprehensive risk assessment regarding treatment procedures is conducted. The patient’s clinical factors and interval from the final ischemic attack are important factors to consider. At our institution, the indication and procedure of treatment are determined through strict preoperative risk assessment. For initial treatment, primary balloon angioplasty alone is performed, and stenting is considered in patients with insufficient dilation, marked acute dissection, or restenosis. We do not recommend stenting as initial treatment for lesions of the MCA or BA involving perforators or branching vessels, which have a high incidence of complications [14].

One of the disadvantages of balloon angioplasty is considered to be more restenosis than stenting. Ueda et al. reported that restenosis was observed in 31.9% of patients who underwent primary balloon angioplasty for symptomatic MCA stenosis with a median follow-up of 63 months [13]. Previous studies reported the incidence of restenosis after balloon angioplasty alone as 24 to 50% [17, 18]. Three recent meta-analyses demonstrated that the rate of restenosis was 8.9% [7], 18.4% [11], and 20% [12].

In the SAMMPRIS trial, the 3-year cumulative incidence of restenosis after stenting for symptomatic ICAS was 14% [19], Furthermore, restenosis was reported to be significantly associated with recurrent ischemic stroke [19, 20]. The Wingspan One-year Vascular Events and Neurologic Outcomes trial demonstrated a relatively low 8.5% 1-year stroke and death rate in Wingspan-stent-treated patients [21]. A meta-analysis demonstrated that the incidences of restenosis in the United States and Asia were 28.8 and 13.6%, respectively, demonstrating a regional difference [22]. Furthermore, this meta-analysis demonstrated that younger age was related to high in-stent restenosis rates [22]. A systemic review of symptomatic intracranial stenting reported that the rate of restenosis was 15.6% [23]. However, risks factors of restenosis after endovascular treatment for symptomatic intracranial stenosis have not yet been established.

A recent randomized trial reported that compared with bare metal stents (BMS), drug-eluting stents (DES) reduced the risks of in-stent restenosis (ISR) and ischemic stroke recurrence in patients with symptomatic high-grade ICAS [24]. Another randomized trial found that the use of a DES compared with a BMS resulted in a decreased risk of ISR, a similar rate of successful implantation of the stent, and similar adverse events [25]. A small single-center study reported that drug-eluting balloons may be a promising alternative treatment for patients with symptomatic high-grade ICAS showing a significantly lower rate of ischemic stroke recurrence or ISR in comparison with the Wingspan stent-treated patients with a similar safety profile [26].

In this study, the overall incidence of restenosis was 26.3% and 5.8% per year. Multivariate Cox regression analysis showed that DM, length of the lesion, and balloon angioplasty were independent predictors for restenosis. DM was reported to be an independent relevant factor for restenosis and recurrent ischemic events after balloon angioplasty alone for symptomatic MCA stenosis [13]. Several studies reported that DM was a predictive factor for restenosis after balloon-mounted stenting for ICAS [27, 28]. For percutaneous coronary intervention (PCI) for coronary lesions, DM is an important predictive factor for restenosis [29]. Neointimal hyperplasia at the lesion site may be primarily involved in the pathogenesis of restenosis. DM promotes neointima formation after PCI [30]. The following clinical factors related to restenosis after PCI have been reported: vascular diameter, lesion length, stent length, and degree of residual stenosis [31]. These characteristics may also be similar for endovascular treatment for intracranial artery stenosis.

This study has several limitations. First, this was a retrospective study at a single center; thus, there was a bias in patient/treatment selection and no control medical arm. Our study design did not compare the superiority or inferiority of treatment outcomes in the percutaneous transluminal angioplasty and stent groups. In the future, a randomized controlled trial with multiple large-volume centers is needed to demonstrate whether this combination therapy is superior to aggressive medical treatment. Second, the results were obtained from a small number of Japanese patients, which may limit the generalizability of our results. Third, the follow-up data on clinical events or images were limited and incomplete data were included.

Endovascular treatment for patients with symptomatic arteriosclerotic ICAS may have potential of better clinical outcome if patients properly selected and treated by an experienced neuro-interventionalist at a high-volume center. However, our results do not allow us to define a profile for patients with this disease to choose between medical treatment and endovascular treatment. Furthermore, well-designed randomized controlled trials are needed to determine the optimal treatment for symptomatic ICAS and the patients targeted.

## Conclusions

A combination therapy of balloon angioplasty and stenting for patients with symptomatic arteriosclerotic intracranial artery stenosis is a feasible procedure with a low incidence of perioperative complications. It may have the potential for better initial and long-term outcomes for appropriately selected patients. Significant restenosis-associated factors were balloon angioplasty and DM. Additional prospective and randomized trials are required to confirm the efficacy of this treatment for recurrent ischemic strokes.

## Data Availability

The data of this study are available from the corresponding author upon reasonable request.

## Abbreviations

ICAS: Intracranial stenosis
MCA: Middle cerebral artery
ICA: Internal cerebral artery
VA: Vertebral artery
BA: Basilar artery
TIA: Transit ischemic attack
mRS: Modified Rankin scale
DM: Diabetes mellitus
DAPT: Dual antiplatelet therapy
SAPT: Single antiplatelet therapy
HR: Hazard ratio
PCI: Percutaneous coronary intervention
ICAS: Intracranial artery stenosis
